# Use of Accelerometers to Monitor Motor Activity During HABIT-ILE for Chronic Stroke: An Exploratory Study

**DOI:** 10.3390/s25216656

**Published:** 2025-10-31

**Authors:** Merlin Somville, Zélie Rosselli, Edouard Ducoffre, Massimo Penta, Tristan Smeesters, Yannick Bleyenheuft, Geoffroy Saussez

**Affiliations:** 1 Motor Skill Learning and Intensive Neurorehabilitation Lab (MSL-IN Lab), Institute of NeuroSciences, UCLouvain, 1200 Bruxelles, Belgium; merlin.somville@uclouvain.be (M.S.); zelie.rosselli@uclouvain.be (Z.R.); saussezg@helha.be (G.S.); 2 Forme & fonctionnement Humain (FfH) Lab, CeREF-Santé, Haute Ecole Louvain en Hainaut (HELHa), 6061 Montignies-sur-Sambre, Belgium; ducoffree@helha.be; 3 Institute of NeuroScience, UCLouvain, 1348 Louvain-la-Neuve, Belgium; massimo.penta@uclouvain.be; 4 Arsalis SPRL, 1473 Glabais, Belgium; tristan@arsalis.com

**Keywords:** IMU, accelerometer, sensors, chronic stroke, intensive rehabilitation, HABIT-ILE, motor skill learning, bimanual therapy, motor function

## Abstract

**Highlights:**

**What are the main findings?**

- Individuals with stroke showed high inter-individual variability in their upper and lower extremities activity pattern throughout the intervention.
- Individuals with a moderate to high dexterity baseline showed a trend for similar evolution of their activity pattern on the upper extremities during a 2-week HABIT-ILE intervention.

**What is the implication of the main finding?**

- Defining common motor activity patterns in individuals with stroke with moderate to high upper extremity dexterity baseline seems possible.
- Developing and implementing such robust protocols in a large number of individuals with stroke would allow defining clear patterns for different profiles of individuals with stroke.
- Defining patterns of treatment based on the characteristics of the patients should help therapists to optimally adapt their treatment content and improve the specificity and efficiency of their interventions.

**Abstract:**

(1) Background: Hand and Arm Bimanual Intensive Therapy Including Lower Extremities (HABIT-ILE) is a high-dose evidence-based neurorehabilitation. This study aims to develop and validate a protocol using three inertial measurement units (IMUs) to objectively document upper and lower extremities’ (UE; LE) motor activity during a HABIT-ILE intervention in chronic stroke adults. (2) Method: Thirteen adults (57.1 y ± 11.3) who completed 65 h of HABIT-ILE (2 weeks, 6.5 h/d) were included. Daily motor activity was recorded with IMUs placed on both wrists and one thigh with nine IMU-derived variables extracted to evaluate motor activity and posture. Each variable was correlated with baseline abilities and day-to-day patterns were observed with subgroup analyses based on baseline dexterity and walking endurance. Day-to-day patterns were highlighted based on mean values and effect size analyses. (3) Results: Only the Magnitude and Use ratios showed high correlations with baseline abilities, with a day-to-day specific pattern highlighted for participants with moderate to good dexterity at baseline. (4) Conclusions: All participants reported a high level of engagement during HABIT-ILE independently of their baseline abilities. Although we could not detect a global trend to document the content of a HABIT-ILE intervention, these exploratory results suggest IMU monitoring to be relevant to characterize therapeutic content.

## 1. Introduction

Stroke is one of the leading causes of motor disability in adults and often results in chronic motor, sensory, and cognitive impairments that affect the activities of daily living and quality of life [1,2]. Arm hemiparesis persists in 55 to 75% of stroke survivors, significantly affecting the ability to perform daily activities [3]. Rehabilitation plays a key role in reducing the impact of these impairments and improving individuals’ quality of life. Among various rehabilitation strategies, interventions based on motor skill learning principles are strongly recommended [4,5]. Notably, Constrained Induced Movement Therapy (CIMT) demonstrated significant improvements in upper extremity (UE) function and activity performance [6]. However, CIMT emphasizes unimanual training, sometimes perceived as frustrating and restrictive by individuals with stroke, which may reduce adherence [7]. Moreover, the initial protocol does not include lower extremity (LE) training, and modified versions targeting LE were not superior to conventional treatment in the long-term [8].

More recently, the Hand and Arm Bimanual Intensive Therapy Including Lower Extremities (HABIT-ILE) was developed, using the effective elements of CIMT with a focus on bimanual coordination training alongside a constant lower extremity and postural control stimulation [9]. Its efficacy in improving LE and UE functions is well-established in children with unilateral cerebral palsy [10] and currently under investigation in adults with stroke [11,12].

Although HABIT-ILE principles are well-described in the literature and follow motor skill learning principles, maintaining an accurate documentation of the individual therapeutic content of such intervention remains a challenge (type of movements performed, upgraded difficulty, type of hand/upper extremities use, etc.). Every therapeutic session is different for every individual with stroke, with a daily adaptation of the treatment plan based on the baseline abilities, preferred strategies, and the specificity of the self-determined goals set to guide the therapeutic program. As the progressive increase in difficulty is not based on a predefined pattern (e.g., specific progression on ankle and knee flexion for a patient willing to go upstairs vs. a progressive decrease in the size of objects manipulated for a patient willing to button his shirt), less experienced therapists may encounter difficulties in foreseeing the next steps of the intervention. Therefore, identifying specific patterns regarding either the baseline abilities or the goals identified could be of tremendous help in generalizing such interventions. Activity diaries (self-reported or not) are commonly used to document the type of therapeutic activities performed during the intervention. However, they are limited by recall bias and inaccuracy, notably regarding the number of performed movements [13].

In this context, wearable inertial measurement units (IMUs), including tri-axial accelerometers, might offer an alternative objective tool to quantify and qualify movement performance during such high-dose interventions [14,15]. Because they are lightweight, relatively low-cost, and capable of handling continuous data over extended periods of time, IMUs facilitate real-world movement monitoring [16]. Although IMU signals alone do not reveal the specific actions performed, they provide a more reliable and objective estimate to track and compare what kind of activity is performed and how much the individuals with stroke are active during therapy [17,18].

**Figure 1:**
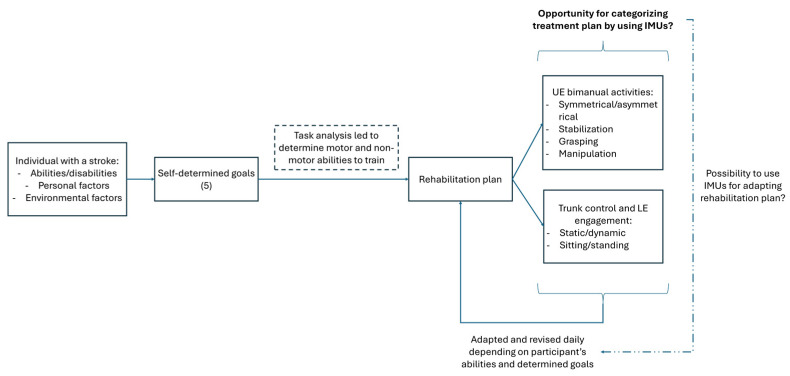
Directed acyclic graph illustrating the hypothesized relationships between baseline motor abilities, IMU-derived variables, and observed motor activity during HABIT-ILE therapy. The diagram summarizes the conceptual framework supporting the use of wearable IMUs to characterize motor activity. UE = upper extremity, LE = lower extremity.

The primary objective of this study is to explore correlations between baseline clinical measures and IMU variables to identify relevant baseline measures to classify functional abilities of individuals with stroke. Second, we aim to identify clinically relevant IMU-derived parameters that effectively monitor and quantify therapeutic activity on both the upper and lower extremities in stroke adults. These advancements are expected to increase the precision of the monitoring of therapeutic content during such high-dose interventions and potentially highlight some specific patterns of treatment characteristics depending on individuals baseline abilities to help therapists in the future to build rehabilitation programs based on individuals with stroke activity profiles (Figure 1).

## 2. Materials and Methods

### 2.1. Ethics

The study was approved by the Hospital-Faculty Ethics Committee of Saint-Luc–UCLouvain, Belgium (clinical trial number: B4032022000142). Individuals with stroke were informed through a comprehensive document and signed an informed consent form.

### 2.2. Study Design

This study is a longitudinal exploratory study that is part of a larger project and was registered on clinicaltrial.gov (NCT05727111).

### 2.3. Participants

Participants were recruited from Belgium and surrounding countries through mailing, spontaneous applications, social networks, physicians, physiotherapists, occupational therapists, and hospitals. All participants were recruited with the following inclusion criteria: (1) a diagnosis of chronic stroke (more than six months post-stroke), (2) be at least 18 years old at inclusion, (3) have the ability to interact and understand simple instructions to complete assessments and therapy, (4) have the ability to perform an onset of movement with the upper extremity. The exclusion criteria were (1) botulinum toxin injections, orthopedic surgery, or another intensive therapy during the 3 months prior to the first assessment or during the study period, (2) be pregnant, (3) have uncontrolled seizures, and (4) have other uncontrolled health issues such as heart or renal failure. Individuals with stroke included in this study were all included in the HABIT-ILE on-site intervention, a 65 h over 2 weeks HABIT-ILE intervention carried out in a rehabilitation center.

### 2.4. Procedure

**Figure 2:**
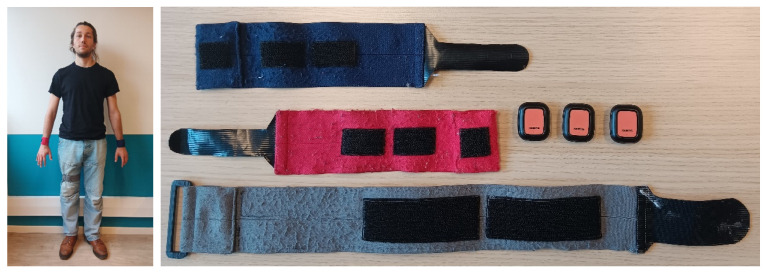
On the **left**: sensor positioning on an individual with stroke. On the **right**: hook and loop straps and three sensors: red on the right wrist, blue on the left wrist, and grey on the right thigh.

Each individual with stroke wore 3 IMUs (Movella Xsens DOT, Xsens Technologies, Enschede, The Netherlands) attached with hook and loop straps at the beginning of each day of the HABIT-ILE intervention. Two were placed dorsally on the wrists and one on the front of the right thigh (Figure 2). Movella DOTs are IMU-equipped with a 3-axis accelerometer, gyroscope, and magnetometer in a compact (36 × 30 × 11 mm), lightweight (11.2 g), and waterproof box. In the present study, we analyzed only the 3-axis accelerometer data. The sampling frequency was set to 30 Hz. The amount and type of motor activity was assessed daily during each of the 6.5 h of therapy, over 10 weekdays (2 weeks, weekends excluded). At the end of the day, IMUs were removed and charged, and data were exported.

Each individual with stroke received 65 h of a HABIT-ILE on-site intervention. HABIT-ILE was delivered for 6.5 h/day for 10 consecutive weekdays with 3.5 h in the morning, 3 h in the afternoon, and a 1.5 h resting/nap time in between. HABIT-ILE is a motor skill learning-based intervention, focusing on both the bimanual coordination and concomitant stimulation of trunk control and lower extremities’ abilities [9]. The performed activities are goal-directed, including an intensive practice of task-oriented enjoyable activities with an increasing difficulty and a “hands-off” practice. Each participant receives an individualized intervention with one or two dedicated interventionists. The therapeutic plan is supervised and adapted on a daily basis by a supervision team. More information about HABIT-ILE intervention is available in the previously published papers [9,11].

### 2.5. IMU Raw Data Treatment

**Figure 3:**
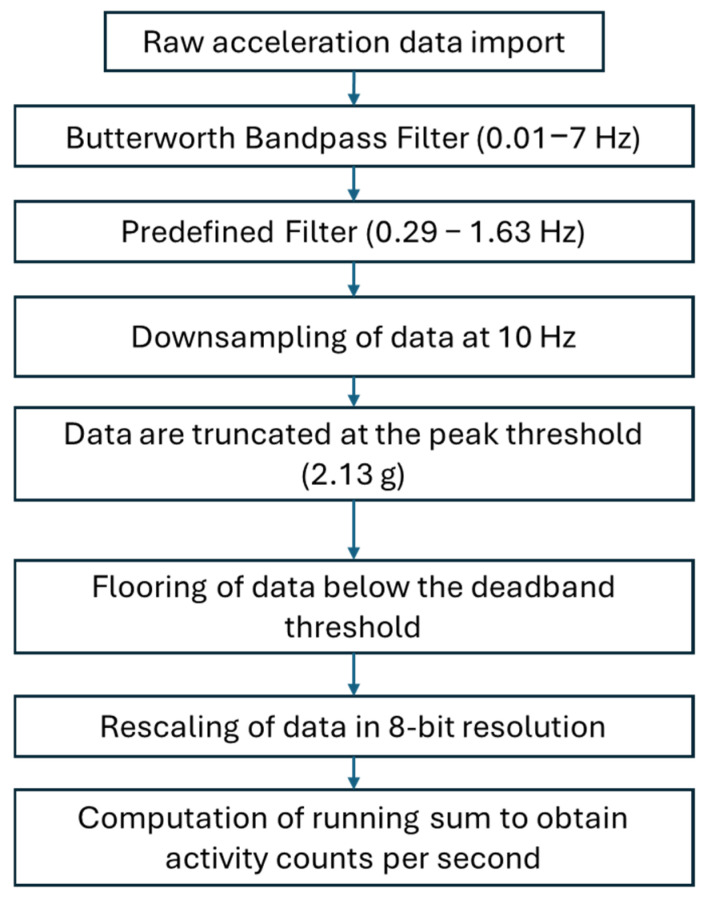
Schematic diagram of the raw IMUs’ data pipeline to obtain activity counts. Hz = Hertz, g = 9.8 m/s^2^.

Raw accelerometry data were processed using custom Python software (Python 3.11.11) adapted from a MATLAB script, previously described by Brønd et al. [19]. First, the acceleration data from each axis (x, y, z) were filtered using a Butterworth bandpass filter (0.01–7 Hz). Subsequently, an additional filtering step using a predefined filter was applied to refine the signal within a passband range of 0.29–1.63 Hz [19]. In the third step, the data were downsampled to 10 Hz. Fourth, values exceeding a predefined peak threshold (±2.13 g) were truncated, effectively capping the extreme values. Fifth, data points below an adapted deadband threshold (0.042 g) were truncated to zero. In the sixth step, absolute values were converted to an 8-bit scale with a resolution of 0.0164 g, covering the range from 0 to 2.13 g. Subsequently, a running sum was computed to derive activity counts per second, resulting in a unitless value that reflects the intensity of movement during that second. The activity counts from each axis were combined into a single resultant value for each one-second interval using the vector magnitude calculation (√(x^2^ + y^2^ + z^2^)) (Figure 3). The same steps were applied for LE with a deadband value of 0.018 g.

The lower deadband threshold for activity detection on both the UE and LE was determined based on videos of individuals with stroke performing activities while wearing IMUs. The videos were independently observed by two assessors to determine periods of activity and inactivity of each extremity equipped with an IMU [20]. The videos were synchronized with the IMU recordings within a time difference not exceeding 33 ms, and both signals were used to determine the optimal threshold to detect motor activity using an ROC curve [17] (See Supplementary S1). For further analysis, data were cleaned for invalid recordings and summarized for each individual with stroke and for each day. To eliminate instances where IMUs were not worn, any period of inactivity lasting at least 5 min was excluded. To ensure a reliable estimation of the activities performed each day, records were only included in the analysis if they lasted more than 9000 s.

### 2.6. Outcome Measures

Nine IMU-derived variables were calculated to quantify and qualify motor activity during each day of HABIT-ILE intervention. Six variables were calculated based on the IMUs worn at the wrists:

(1) The percentage of use for each of the UEs represents the percentage of time where the UE activity (wrists) is above the threshold to detect motor activity during the daily recorded total time.

$$Percentage\;of\;use\;of\;the\;UE=\frac{Active\;time}{Total\;time\;of\;the\;record}\times 100$$

(2) Activity magnitude (AM) measures the intensity of the detected movements for each of the UEs, expressed in counts per second (counts/s).
(3) The bimanual use (BU) represents the percentage of motor activity time, where a bimanual activity is recorded by the IMU in the daily recorded total active time.

$$Bimanual\;use=\frac{Time\;with\;bimanual\;use}{Total\;Time}\times 100$$

(4) Bilateral magnitude represents the intensity of detected movements in both of the UEs. It is calculated by summing the AM of both of the UEs for each second of activity, expressed in counts/s [17].

Bilateral Magnitude=Activity magnitude affected UE+Activity Magnitude Non affected UE

(5) The use ratio is calculated by dividing the percentage of use of the affected UE with the one of the non-affected UE. A ratio > 1 expresses a higher percentage of time with movement recorded on the affected UE. A ratio < 1 expresses a higher percentage of time with movement recorded on the non-affected UE.

$$Use\;Ratio=\frac{Time\;with\;use\;of\;affected\;UE}{Time\;with\;use\;of\;non\;affected\;UE}$$

(6) The magnitude ratio represents each UE’s contribution to the movement. Its calculation has been performed based on the methodology described by Bailey et al. (2014) [17]: one activity count is added to the smoothed vector magnitude of both UEs, the smoothed vector magnitude of the non-dominant UE is then divided by that of the dominant UE, and the resulting values are finally log-transformed by natural logarithm. Values range from −7 to 7, where 0 indicates an equal contribution of both UEs, with positive values indicating a higher contribution of the affected UE and negative values a higher contribution of the non-affected UE [17].

$$Magnitude\;Ratio=\ln\frac{Activity\;magnitude\;of\;affected\;UE}{Activity\;magnitude\;of\;the\;non\;affected\;UE}$$

The percentage of bimanual use, bilateral magnitude, and ratios were calculated based on treatment periods with effective recordings obtained for both UEs at the same time.

The last three variables were calculated based on the IMU worn on the right thigh:

(7) The percentage of use of the LE represents the percentage of time where a movement is recorded by the IMU worn on the right thigh during the daily recorded total time.

$$Percentage\;of\;use\;of\;the\;LE=\frac{Active\;time}{Total\;time\;of\;the\;record}\times 100$$

(8) The activity magnitude measures the intensity of the movements detected at the thigh of the right LE, expressed in counts per second (counts/s).
(9) The positioning of individuals with stroke, expressed in percent of the daily total recorded time, are calculated based on the orientation of the IMU worn at the thigh. Based on a simple vertical 45° threshold, the positioning was determined to be standing (>45°) or sitting/lying (<45°).

### 2.7. Baseline Functional Performance

Baseline motor abilities were documented using two assessments. The Box and Blocks Test (BBT) measured gross unimanual dexterity, defined as the number of blocks moved in one minute [21]. The walking endurance was evaluated with the 6 Minute Walking Test (6MWT), during which participants walked as far as possible in six minutes along a 30 m hallway without resting [22].

### 2.8. Data Analysis

Statistical analyses were performed using Rstudio software (version 2023.12.15+402, Posit Software PBC, Boston, MA, USA) with R version 4.4.3. Before conducting analyses to document treatment content, we tested the correlation between baseline performances, captured by the BBT and 6MWT, and the mean values of each IMU-derived variable using Spearman’s rank correlation. This step aimed to verify the hypothesized relationship between these baseline measures and the IMU derived variables and to confirm their relevance for creating subgroups to document rehabilitation plans and activities.

To explore day-to-day patterns during the 2-week HABIT-ILE intervention, each participant’s daily mean value was first calculated for every IMU-derived variable. The daily means were then averaged to produce a global pattern for each day across the two weeks of intervention. Subsequently, a day-to-day pattern was observed, based on the effect size calculation, to document the magnitude of change across the intervention days. The daily effect size was calculated with the following formula:

$$Effect\;Size=\;\frac{Daily\;Mean-First\;Day\;Mean}{\begin{array}{c} Average\;standard\;deviation\;of\;the\;variable\;observed\\ for\;the\;participant\;over\;all\;therapy\;days \end{array}}$$

The resulting effect sizes were then averaged day by day to generate a global curve.

All analyses were conducted through subgroups based on participants’ baseline abilities. For the UE, 3 subgroups were defined by the BBT score: *n* = 0 was described as “very low UE motor function” (unable to perform the test), “low UE motor function” was defined by a BBT Z-score ≤ −5, and “medium to good UE motor function” was defined by a BBT Z-score > −5. Z-scores were calculated based on the normative values described by Mathiowetz et al. [21]. For the LE, participants were classified into two subgroups according to 6MWT performance, using a cutoff of 286 m (≤286 m or above) (all participants were able to perform the test). This cutoff is considered as the minimal threshold for a functional ambulation [23].

## 3. Results

Fourteen individuals with stroke who participated in a HABIT-ILE intervention were equipped with IMUs. In these 14 individuals, one dropped out of the study after the first day of therapy due to the logistical burden of transportation to/from the rehabilitation center. Data analysis for 13 participants was included in the present study. After raw data processing and treatment, 74 to 90 therapeutic days (depending on variables) were available for analysis out of the 130 therapeutic days performed. Demographic characteristics and descriptive analyses of the 13 participants are displayed in Table 1 and Table 2.

During the 2 weeks of therapy, IMUs detected movements for around 51% of the time for the affected UE and 67% for the non-affected UE, with a mean activity magnitude around 25 counts/s for the affected UE and 57 for the non-affected UE. Individuals with stroke showed bimanual activities for around 43% of the time with a bilateral magnitude (BM) around 81 counts/s. The use ratio (UR) and magnitude ratio (MR) showed a larger use of the non-affected arm with respective values of 0.75 and −1.91. Individuals with stroke performed movement with the LE for around 48% of the time, with around 28% of the time in a standing position and 72% sitting/lying.

### 3.1. Correlations Between Baseline Abilities and IMUs-Derived Variables

**Figure 4:**
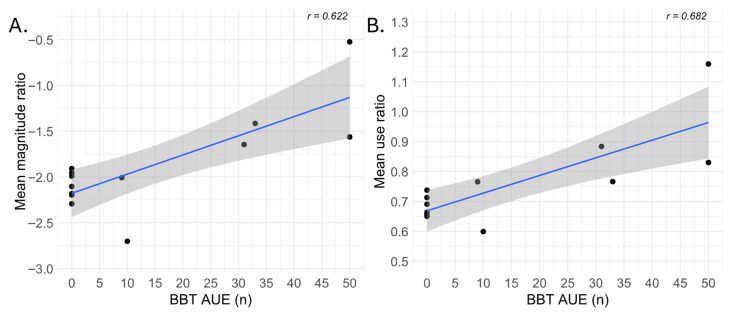
**Spearman correlations between IMU-derived variables and Box-and-Block Test score at baseline for the affected upper extremity.** (**A**) Mean magnitude ratio: values vary between −7 and +7, where positives values indicate higher magnitude of the affected arm. (**B**) Mean use ratio: values above 1 indicate a higher % of use of the affected arm. Each black dot represents one individual with stroke mean value over the 10 days of intervention (n = 13). The solid blue line is the regression line; the shaded grey band represents its 95% confidence interval. r = Spearman’s rank-correlation coefficient. AUE = affected upper extremity; BBT = Box and Block Test.

The Pearson correlation coefficients between the baseline abilities and IMUs-derived variables showed non-significant correlations for all the tested variables (r ≤ |0.38|, *p* ≥ 0.20), except for the baseline scores of the BBT and the values observed for the use ratio (r = 0.68, *p* < 0.05) and magnitude ratio (r = 0.62, *p* < 0.05) (Figure 4). The results of all the correlations are displayed in the Supplementary Material S2.

### 3.2. Day-to-Day Patterns for Upper Extremities’ IMU-Derived Variables

**Figure 5:**
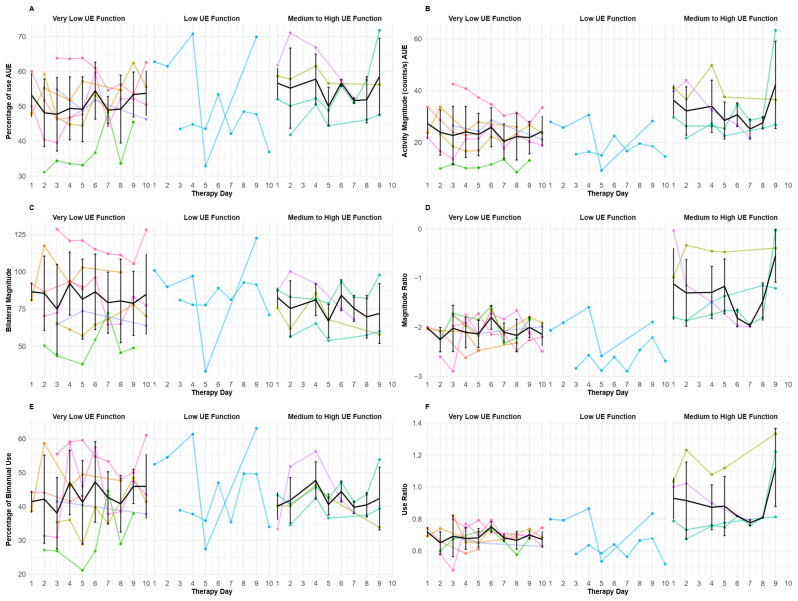
**Mean day-by-day IMU-derived variables across therapy days for individuals with stroke.** Each panel corresponds to one IMU-derived affected upper extremity (AUE) variable throughout the 10 days of therapy; in each panel, the three groups based on BBT are shown. The colored lines correspond to each stroke survivor measurement, the black line corresponds to the mean evolution curve when taking all the records available for each day, and the standard deviation is shown for the mean evolution curve. (**A**) represents the evolution of the percentage of use of the AUE, (**B**) represents the activity magnitude of the AUE, (**C**) represents the bilateral magnitude, (**D**) represents the magnitude ratio, (**E**) represents the percentage of bimanual use, and (**F**) represents the use ratio. The global curve was not represented for the low UE motor function group, because only two participants were in this group.

The mean IMU-derived day-to-day values for upper extremities are represented in Figure 5. For all IMU-derived variables, in each subgroup, no general pattern seems to emerge throughout the days of therapy except for the magnitude ratio and use ratio in the medium to good UE function (Figure 5D,F). For these participants with moderate-to-good UE function, although high inter-individual variability was observed, the magnitude and use ratios looked generally stable during the first week, seemed to slightly decreased at the beginning of week 2, and increased again by the end of the intervention.

**Figure 6:**
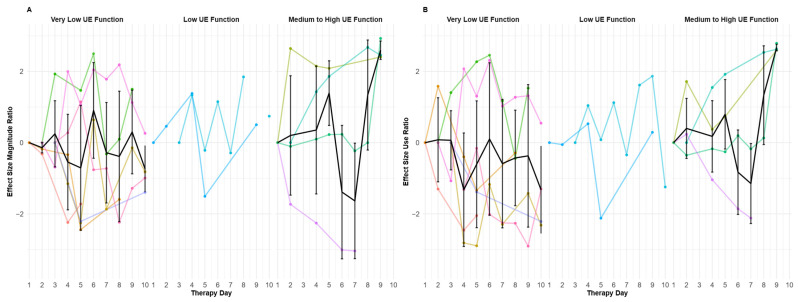
**Day-to-day effect size changes in IMU-derived variables during 2-week HABIT-ILE interventions.** Effect size is calculated for each participant over all therapy days. The black line represents the averaged day-to-day effect sizes, with standard deviation, for all participants. Colored lines correspond to each stroke survivor effect size variable measurement. (**A**) represents the changes in magnitude ratio, and (**B**) represents the change in use ratio.

To document the magnitude of change across intervention days, a mean effect size was calculated for each participant. As described previously (Figure 4), only the magnitude and use ratios showed significant correlations with the baseline dexterity level. Thanks to these correlations and Figure 5 showing more noticeable patterns for the moderate to good functioning participants, effect size analyses were only conducted for these two variables (Figure 6). When displayed with the effect size calculation, the observed pattern seems similar to that previously observed in Figure 5. In the subgroup with very low UE function, the magnitude and use ratios appear stable throughout the 10-day therapy period (with a slight decrease over the 10 days) (Figure 6A). In the subgroup with the low UE function, it is difficult to discriminate a pattern due to the small number of participants. In the medium-to-good UE function subgroup, the magnitude ratio seems quite stable during the first week (days 1 to 5), decreases at the beginning of the second week, and then increases during the last days of the HABIT-ILE intervention (Figure 6A). The use ratio appears to follow a similar pattern with a less pronounced decrease at the beginning of the second week (Figure 6B).

### 3.3. Determining Day-to-Day Patterns for Lower Extremities’ IMU-Derived Variables

**Figure 7:**
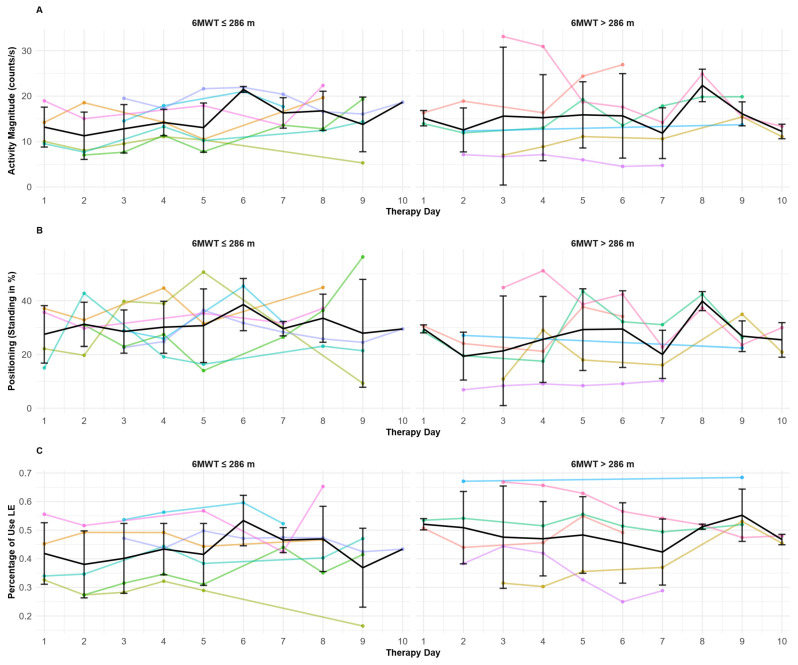
**Lower extremities’ IMU-derived variables across therapy days.** (**A**) represents the activity magnitude of the right lower extremity (LE), (**B**) represents the percentage of time spent in a standing position, and (**C**) represents the percentage of use of the right LE. For each panel, the two subgroups are shown based on 6MWT baseline abilities. The colored lines correspond to each stroke survivor measurement, and the black line corresponds to the mean day-to-day evolution curve. The standard deviation is shown for the mean evolution curve.

Figure 7 displays the IMU-derived variables for the lower extremities, and no clear day-to-day trend was observed. Across the 10 days of therapy, the variables remained relatively stable, with no marked differences between the two subgroups defined based on the 6MWT performance.

## 4. Discussion

In this exploratory study, we focused on characterizing activities performed during HABIT-ILE, with data available for 13 individuals with stroke. Spearman correlations revealed that the use ratio and magnitude ratio displayed moderate significant correlations with the baseline BBT score of the affected hand. No specific pattern of motor activity seems to emerge during the two weeks of HABIT-ILE when using IMU-derived variables, except for the use ratio and magnitude ratio in individuals with moderate to high dexterity abilities in the more-affected UE at baseline. However, HABIT-ILE seems able to stimulate the high motor engagement of all participants. These results will be discussed in the following sections.

Comparing IMU-derived metrics across studies remains challenging, because each manufacturer applies proprietary signal-processing pipelines to transform raw tri-axial acceleration data into activity counts. Consequently, any direct comparison should be interpreted with caution, as the details of these proprietary processing algorithms are not always disclosed [16]. However, examination of the IMU-derived activity across different studies seems valuable for contextualizing the type and intensity of practice achieved in different interventions or during daily living. The study by Bailey et al. (2015) used a similar methodology for the calculation of variables, recording 24 h of daily routine activities in chronic stroke and demonstrated that the affected upper extremity (AUE) was active for 4.9 h per day (unilateral + simultaneous bilateral activity), representing around 20% of the 24 h period [18]. Because the original study did not report the sleep duration, subtracting the mean sleep time of older adults (7.4 h) increases the active time of the AUE to approximately 29% of waking hours [24]. During HABIT-ILE, the AUE was engaged for 51% of the session, suggesting that HABIT-ILE enhances AUE involvement during therapy. For bilateral magnitude and magnitude ratio, the results observed during the HABIT-ILE intervention were comparable between daily routine activities and HABIT-ILE [18,25]. Nonetheless, HABIT-ILE seems to also improve bimanual use; whereas Bailey et al. (2015) documented 4.1 h/day of bimanual use (around 25% of waking hours), individuals with stroke in the HABIT-ILE group achieved 43% of bimanual use during therapy sessions [18]. These findings suggest that, although HABIT-ILE increases AUE engagement, it does not increase the overall movement magnitude. For the thigh-mounted IMU, a 24 h daily living record indicated that individuals with stroke spent 9% of the time standing and 7% walking [26]. If we account for the same sleep duration as inferred for AUE, it would be equivalent to around 22% of the waking time, indicating that the 6.5 h of a HABIT-ILE did not markedly increase the standing duration, with a standing duration of 28% of therapy time. A second study reported a 12% walking and 19% standing time, reinforcing this observation that the standing duration was not increased [27]. Nonetheless, the activity performed while standing probably differs between HABIT-ILE sessions and daily routine, with more challenging activities performed during therapeutic time rather than static or less demanding standing activities at home. Moreover, it is important to note that sitting during HABIT-ILE is not sedentary: therapists continuously challenge the trunk and lower extremities (e.g., sitting on inflatable ball or benches), and many “sitting” tasks include dynamic components such as lateral displacements along the bench. Although this interpretation is based on clinical observation rather than direct measures of trunk or muscle activity, it reflects the structured therapeutic design of HABIT-ILE, where sitting tasks systematically involve active postural control. Thus, HABIT-ILE sitting fundamentally differs from passive sitting in daily life. Consistent with this interpretation, despite sitting accounting for approximately 72% of the recorded time, lower-limb activity was detected for 47% of that period.

Correlations between the mean values of the IMUs-derived variables for each individual with stroke and their baseline upper and lower extremities abilities were weaker than expected. Only two parameters, the use and magnitude ratios, showed a significant correlation with baseline BBT scores. The generally weak or non-significant correlations observed for most variables suggest that either the chosen IMUs setup is not the most suitable for documenting the treatment content of a HABIT-ILE intervention, or the selected variables are not the most appropriate for characterizing the content of therapeutic sessions performed during HABIT-ILE intervention. Regarding the aim of characterizing the content of the HABIT-ILE intervention, for UEs, a trend for activity patterns in use and magnitude ratios throughout the 10-day intervention seemed to emerge only in individuals with stroke with moderate to high baseline UE motor function. Among these higher-functioning individuals with stroke, the use ratio and magnitude ratio remained relatively stable during the first week, a period dedicated to training new motor skills. Because these movements were difficult but achievable, and the task difficulty progressively increased as new motor skills emerged, the parameters likely remain relatively stable. At the start of the second week, most individuals with stroke began to include the newly learned abilities into partial or full components of their functional goals. This phase can lead to a slight decrease in the use ratio and magnitude ratio due to the new tasks demands and needs (e.g., specific spatial organization, praxis, …) [28]. This initial decrease in the use ratio and magnitude ratio was then followed by a secondary increase likely linked to higher abilities and a more fluent use of the affected arm, which allowed a higher number of repetitions and more fluid movements [29]. By the end of the two-week HABIT-ILE program, self-determined goals were practiced more intensively and in varied contexts to improve real-life transfer, further increasing the use ratio and magnitude ratio [11]. Despite the absence of a clear trend throughout the 10 days of intervention in individuals with lower UE motor function, all participants appeared actively engaged in therapy, with movement of the more-affected UE detected for at least 50% of therapeutic time. This finding underlines that even individuals with stroke and low motor function are able to successfully actively engage in HABIT-ILE therapy, whereas in some other intensive intervention (e.g., CIMT), minimal motor inclusion criteria might restrain these patients from participating [30]. Moreover, this engagement seems stable over the 10 days of intervention.

Despite the results observed in the use ratio and magnitude ratio, the other analyses led on the upper extremities variables raise questions about the possibility of categorizing such therapeutic content and about the best way to do so in terms of material (placement, type of technology) and baseline variables for a predictive rehabilitation plan. First, the lack of trends detected in individuals with stroke with more limited upper extremity abilities reflects the lower reliability of IMU measurement for individuals with stroke with more severe motor impairment [31]. For these individuals with stroke, the trained abilities for the upper extremities may involve basic grasp control, with minimal arm displacement, or an active static-stabilization role of the hand. Because these behaviors generate little or no acceleration at wrist level, they are not identified as activity or as bimanual activity by IMUs [32]. Consequently, IMUs tend to under-detect the trained abilities for these individuals with stroke. Additional measurements such as EMG or structured video analysis may capture these changes more accurately [32,33]. Moreover, as accelerometers for the upper extremity were only positioned at the wrists, it is impossible to capture compensatory movement occurring at the shoulder or trunk during therapy [28]. This could also at least partly explain the difficulty of finding a common pattern in the participants with more severe impairment.

Second, regarding the baseline variables to categorize individuals with stroke, the baseline BBT score showed two significant correlations, corresponding to the two variables showing a trend to observe a specific treatment pattern. Since the other variables showed no correlation or specific treatment pattern, there might be a need for a different more precise baseline categorizing. A baseline analysis focusing on self-determined goals and the specific motor and non-motor features needed to train during the intervention (i.e., task analysis) might increase the prediction for further subgroup analyses.

The results of the LE engagement did not correlate with the IMU-derived variables; without a clear pattern observed during the intervention, this raises similar questioning about the material and the way to categorize individuals with stroke. First, the 6MWT, even if it measures walking endurance, might not be the best outcome to distinguish subgroups and characterize intervention. Indeed, as the exact content of training is tailored based on self-determined goals, a more comprehensive baseline assessment based on specific task analysis might provide more relevant information to characterize the potential therapeutic content. Second, although the thigh-mounted IMU provides reliable and valid data for quantifying LE movements and distinguishing sitting from standing postures [34], it neither captures knee or ankle flexion/extension nor quantifies weight shifting and distribution in static or dynamic situations. Because these elements are part of the frequently trained abilities while focusing on functional goals including LE or UE/LE coordination during HABIT-ILE, a single easy-to-set-up thigh sensor may be insufficient to document the treatment content during such intervention. Future studies might consider adjustments on the LE activity measurement to more precisely document the therapeutic content and LE motor activities, for example, by combining the thigh placement with an additional IMU and pressure sensors under individuals with stroke’ foot.

Finally, among the 10 delivered intervention days, each participant presented 5 to 7 days of usable data. This large data loss could be explained by multiple factors. One first issue was software-related. The Movella Dot application was unstable on launch with several re-launchings needed before effective recording. Similar issues occurred during data saving, where some IMUs were not detected by the computer. Second, the Movella Dot hardware has a power button on the top of the sensor that participants inadvertently pressed during day-to-day movement, causing accidental shut-offs. This had consequences, especially for variables based on the use of both UEs, as the loss of the signal from one of the two IMUs led to the impossibility of calculating these variables. Moreover, despite the IMU nominal battery capacity of 8 h, several units could not sustain continuous recording for the required eight hours daily record (6.5 h of therapy and 1.5 h of rest). This further highlights the need for easy-to-use IMUs adapted to real-world recording, with long-lasting batteries and easy start/stop/export functionality to facilitate data acquisition and export.

### Limitations

As this is an exploratory study, the main limitation of this paper might be the number of participants included. This limitation has been heightened by a larger than expected data loss in IMU recordings. Consequently, all analysis should be interpreted as exploratory, and subgroup comparisons were limited to descriptive observations rather than formal statistical analysis. However, the results observed in this study underline the interest of such IMU recordings for categorizing therapeutic profiles, with adaptations highlighted for more robust and precise measures of the therapeutic content.

Additionally, no baseline IMUs measurements in ecological situations were collected during this study. This could be an interesting alternative for categorizing baseline abilities and potential predictors of the treatment content within a HABIT-ILE intervention.

## 5. Conclusions

This exploratory study is the first to attempt a detailed characterization of therapeutic activities within a HABIT-ILE context with affordable and easy-to-use materials. Analyses reported a high level of engagement during the proposed individualized activities for all participants independently of their baseline abilities. Regarding treatment characterization, the IMU-derived variables showed limited correlations with outcomes at baseline overall. However, the subgroup of individuals with moderate to high dexterity baseline abilities displayed more identifiable therapeutic patterns across the 2-week intervention for the use and magnitude ratios. Future studies might consider enhancing the material used to monitor movement and think about more specifically driven baseline subgrouping based on self-determined goals’ task analysis. This may in the future provide guidance for therapists in the context of treatment plan adjustments.

## Figures and Tables

**Table 1:** Demographic characteristics and baseline abilities.

Age (years)	
mean ± SD	57.1 ± 11.3
Time since stroke (months)	
mean ± SD	30.0 ± 20.7
Gender, n (%)	
Male	6 (46.2%)
Female	7 (53.8%)
Affected side, n (%)	
Right	8 (61.5%)
Left	5 (38.5%)
Type of stroke, n (%)	
Ischemic	10 (76.9%)
Hemorrhagic	3 (23.1%)
mRS, n (%)	
1	1 (7.7%)
2	4 (30.8%)
3	8 (61.5%)
6MWT (m)	
mean ± SD	311 ± 124.1
BBT (n)	
mean ± SD	14 ± 19.7

Data are presented as mean ± SD or number of individuals with stroke and percentage. mRS = modified Rankin scale, 6-point scale from 0 to 6 to assess disability, where a higher score is associated with higher disability. BBT = Box and Block test. 6MWT = Six-Minute Walk Test. n = number of transferred blocks.

**Table 2:** Available data and mean values of IMUs-derived metrics for the 2 weeks of HABIT-ILE intervention.

	Therapeutic Days with Available Data (n/130)	Mean ± SD
Upper Extremities		
Percentage of use (%)		
Non-affected side	*79*	67.3 ± 9.7
Affected side	*84*	51.5 ± 9.2
Activity magnitude (counts/s)		
Non-affected side	*79*	57.3 ± 17.3
Affected side	*84*	25.3 ± 9.6
Bimanual use (%)	*74*	43.4 ± 8.9
Bilateral magnitude (counts/s)	*74*	81.3 ± 21.3
Use ratio	*74*	0.75 ± 0.16
Magnitude ratio	*74*	–1.91 ± 0.59
Lower Extremities		
Percentage of use (%)	*90*	47.6 ± 14.0
Activity magnitude (counts/s)	*90*	17.2 ± 12.0
Standing time (%)	*90*	27.7 ± 14.5
Sitting time (%)	*90*	72.3 ± 14.5

From left to right; the second column indicates the number of therapeutic days during which data were recorded for each metric; the third presents the overall mean ± SD for each metric across individuals with stroke. Values are mean ± SD unless otherwise stated; n indicates the number of therapeutic days contributing to each metric. SD = standard deviation.

## Data Availability

The original contributions presented in this study are included in the article/Supplementary Material. Further inquiries can be directed to the corresponding author.

## Supplementary Materials

The following supporting information can be downloaded at https://www.mdpi.com/article/10.3390/s25216656/s1, S1: ROC curve results; S2: Correlation table. Ref. [35] cited in Supplementary Materials.

## Abbreviations

The following abbreviations are used in this manuscript:

6MWT	6-Minute Walk Test
AC	Activity Count (counts·s^−1^)
ADLs	Activities of Daily Living
AM	Activity Magnitude (counts·s^−1^)
AUE	Affected Upper Extremity
BBT	Box and Block Test
BM	Bilateral Magnitude
BU	Bimanual Use
CIMT	Constraint-induced Movement Therapy
CP	Cerebral Palsy
EMG	Electromyography
HABIT-ILE	Hand and Arm Bimanual Intensive Therapy Including Lower Extremities
Hz	Hertz
IMU	Inertial Measurement Unit (wearable sensor)
LE	Lower Extremity
MATLAB	Matrix Laboratory (MathWorks software (Build 8.6.0.267246 (R2015b) 64 bit).)
MR	Magnitude Ratio
mRS	Modified Rankin Scale
NAUE	Non-Affected Upper Extremity
ROC	Receiver Operating Characteristic (curve)
R^2^	Coefficient of Determination (R-squared)
SD	Standard Deviation
TUG	Timed Up and Go
UE	Upper Extremity
UR	Use Ratio
