# Supplementary material for: Use of Accelerometers to Monitor Motor Activity During HABIT-ILE for Chronic Stroke: An Exploratory Study
Source: Sensors (Basel). 2025 Oct 31;25(21):6656. doi: 10.3390/s25216656 (PMC12610605; doi:10.3390/s25216656)
Supplement: Supplementary file 1 [file sensors-25-06656-s001.zip › S1_ROC curve results.pdf]

To determine optimal threshold, a visual inspection of the video recorded during therapy (3 indivuals with stroke, each with 90-minute record) was performed to encode the presence or absence of movement. This rating was performed independently by two raters using identical rating criteria. For hand movement detection, inter-rater reliability was good, with an ICC = 0.77 [95% CI 0.68–0.84], indicating strong agreement between raters, and a small mean bias (Cohen's d = 0.21 [95% CI 0.12–0.31]), suggesting minimal systematic difference in the number of movements detected.

The ratings were then merged to create a reference data set for evaluating the activity detection algorithm. Receiver Operating Characteristic (ROC) curve was then computed by varying the activity detection thresold value from 0.000g to 0.400g, by 0.002g increment, which maximized the true positive rate (TPR) while minimizing the false positive rate (FPR). The outcomes of this ROC curve are an optimal threshold of 0.042g which maximizes TPR while minimizing FPR and an demonstrated an excellent perfomance of the algorithm charcterized by an area under the ROC curve (AUC) of 0.838 [1] (**Figure S1**).

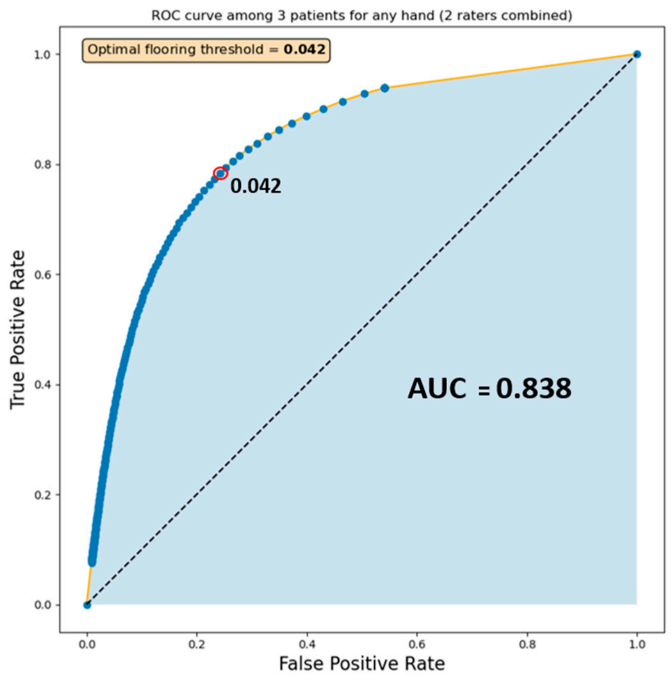

Figure S1. Receiver Operating Characteristic (ROC) curves for activity detection. ROC curve for three stroke patients, with movement annotations provided by two independent raters. The optimal detection threshold (0.042 g) is indicated, and the area under the curve (AUC) is 0.838. Colored dots represent threshold increments, the red circle indicates the optimal threshold, and the shaded area corresponds to the AUC.

The same procedure was applied for lower limb movement detection. For lower-limb movement detection, inter-rater reliability was moderate to good, with an ICC = 0.63 [95% CI 0.42–0.76], indicating acceptable agreement between raters, and a small to moderate mean bias (Cohen's d = 0.38 [95% CI 0.23–0.53]), suggesting that one rater tended to score slightly more movements than the other. An optimal threshold of 0.018 g and an AUC of 0.887, also indicative of excellent

classification performance (**Figure S2**).

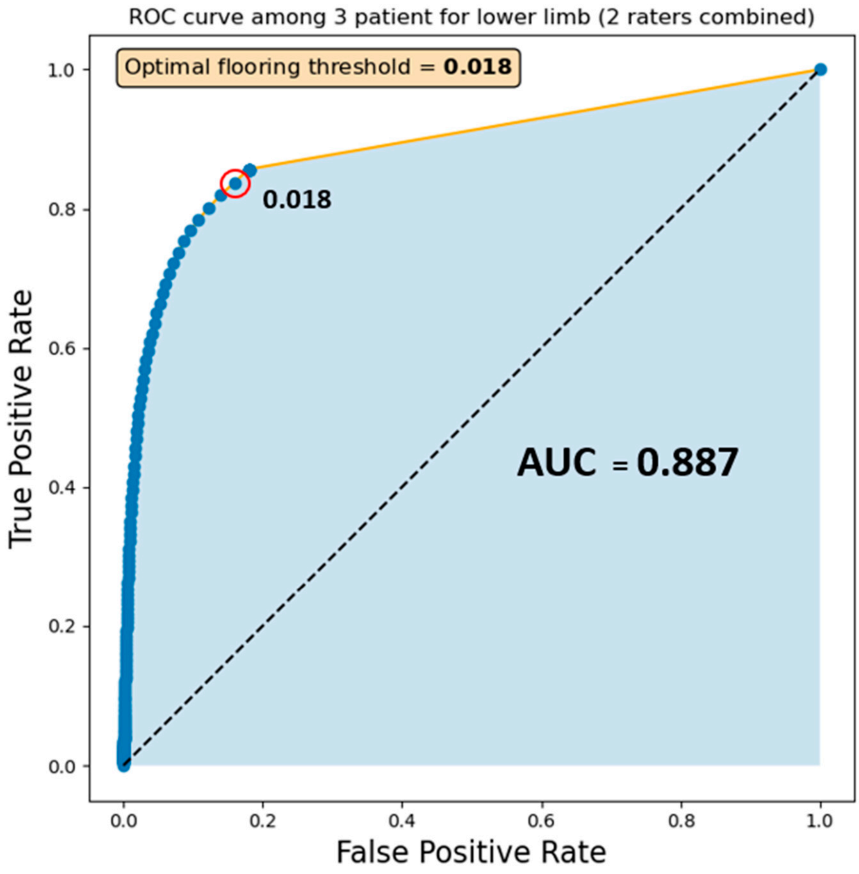

Figure S2. Receiver Operating Characteristic (ROC) curves for activity detection. ROC curve for the lower limb condition, with an optimal threshold of 0.018 g and an AUC of 0.887. Colored dots represent threshold increments, the red circle indicates the optimal threshold, and the shaded area corresponds to the AUC.

TPR = True positive rate

FPR = False positive rate

[1] Mandrekar JN. Receiver Operating Characteristic Curve in Diagnostic Test Assessment
