# Supplementary material for: Use of Accelerometers to Monitor Motor Activity During HABIT-ILE for Chronic Stroke: An Exploratory Study
Source: Sensors (Basel). 2025 Oct 31;25(21):6656. doi: 10.3390/s25216656 (PMC12610605; doi:10.3390/s25216656)
Supplement: Supplementary file 1 [file sensors-25-06656-s001.zip › S2_Correlation_table.pdf]

Table S1. Correlations between IMUs-derived variables and baseline abilities

Variables	Spearman's Correlation	
	R	*p*-Value
Correlations with BBT affected UE		
Percentage of use (%)		
Affected side (%)	0.25	0.41
Activity magnitude (counts/s)		
Affected side	0.38	0.20
Bimanual use (%)	.0.02	0.95
Bilateral magnitude	-0.10	0.74
Use ratio	0.68	**0.01**
Magnitude ratio	0.62	**0.02**
Correlations with 6MWT		
Percentage of use LE (%)	0.38	0.20
Activity magnitude LE (counts/s)	0.05	0.85
Patient positioning (%)	0.13	0.67

AUE = affected upper extremity; NAUE = non-affected upper extremity; BBT = Box and Block Test; TUG = Timed Up and Go test; LE = Lower Extremities; 6MWT = Six-Minute Walk Test; R = Spearman's correlation coefficient. Bold values indicate statistically significant correlations ($p < 0.05$).
